# Free Will, Black Swans and Addiction

**DOI:** 10.1007/s12152-016-9290-7

**Published:** 2016-11-21

**Authors:** Ted Fenton, Reinout W. Wiers

**Affiliations:** 1 King’s College London, Strand, London, UK; 2 Addiction, Development and Psychopathology (ADAPT)-lab, Department of Psychology, Amsterdam Brain & Cognition (ABC), Research Priority Area Yield, University of Amsterdam, Amsterdam, Netherlands

**Keywords:** Addiction, Brain-disease, Bad habit, Free will, Autonomy

## Abstract

The current dominant perspective on addiction as a brain disease has been challenged recently by Marc Lewis, who argued that the brain-changes related to addiction are similar to everyday changes of the brain. From this alternative perspective, addictions are bad habits that can be broken, provided that people are motivated to change. In that case, autonomous choice or “free will” can overcome bad influences from genes and or environments and brain-changes related to addiction. Even though we concur with Lewis that there are issues with the brain disease perspective, we also argue that pointing to black swans can be important, that is: there can be severe cases where addiction indeed tips over into the category of brain disease, but obviously that does not prove that every case of addiction falls into the disease category, that *all* swans are black. We argue that, for example, people suffering from Korsakoff’s syndrome, can be described as having a brain disease, often caused by alcohol addiction. Moreover, the brain changes occurring with addiction are related to choice-behaviour (and the related notions of willed action), habit formation and insight, hence essential mental abilities to break the addiction. We argue for a more graded perspective, where both black swans (severe brain disease which makes recovery virtually impossible) and white swans (unaffected brain) are rare, and most cases of addiction come as geese in different shades of gray.

## Addiction a Brain Disease?

There is no question that addictive behaviours can have negative effects for the individual, including social problems, physical harms and premature death. But that in itself does not imply that it is best described as a brain disease, the current dominant perspective in biomedical sciences, clearly stated by the influential scientist and president of the National Institute on Drug Abuse (NIDA), Nora Volkow [1], in line with her predecessor Alan Leshner in an influential paper in Science [2]. Addiction is related to choices that are (potentially) beneficial in the short-run, at the expense of long-term positive outcomes, a facet of impulsivity known as delay-discounting [3]. However, association does not imply causation, and indeed there is both evidence that impulsive traits can become more pronounced through engaging in addictive behaviours, and that impulsivity is one of the risk-factors for the development of addiction [4, 5]. Increased impulsivity is a first example of brain-changes that can occur with the development of addiction, others include changes in the systems underlying habit [6, 7], salience-processing [8], self-insight [9], and increased negative affect and stress-sensitivity, aptly called “the dark side of addiction” [10]. Most neurobiologically-oriented scientists adhere to a model now including all these different changes in different phases of addiction, where initially there is increased reward and salience of drug-cues (positive reinforcement stage), followed by more automatized habitual responding (compulsive phase), and in later stages by anhedonia and drug use to counter negative effects of previous use (negative reinforcement) [7, 11, 12]. Note that, in addition to different phases, these responses may differ from substance to substance [13], and from person to person, related to personality characteristics [14]. Further, people typically take multiple substances, which may further complicate the exact nature of the brain changes [15]. The question here is whether these brain-changes should be called a brain-disease. In his well-written book and accompanying paper, Marc Lewis argues against this perspective [16, 17]. What are his arguments?

First, Lewis argues that the brain changes all the time, when we learn a new cognitive ability (e.g., a new language), a new skill (e.g., skateboarding), or any new habit. Lewis here cites Doidge: “there’s nothing more fundamental to the human brain than its plasticity”, and adds: “Yet neuroscientists who study addiction seem to have missed the point.” We doubt whether the neuroscientists really missed this point, or merely emphasize that the brain changes are more profound and long-lasting than brain-changes in learning everyday habits. In fact, Lewis agrees that “we must still ask whether there is something special about addiction that makes it hard to overcome,” and then lists “three mechanisms that accelerate our attraction to addictive rewards and entrench addictive activities-without making it a disease.” ( [17], ms. P. 10). This appears to be an empirical question: to what extent brain changes related to substance use disorders are similar to those related to falling in love, or habits involving rewards (gambling, gaming), and there appear to be both commonalities and differences. [18, 19] In fact, parallels in brain functioning in pathological gamblers and people with substance use disorders was an important argument to include pathological gambling in the addictions cluster in the DSM5. Of course, that does not prove that both (or neither) are a brain disease, but does point to a perspective where some brain-changes are more long-lasting and severe than others.

A related point concerns the question to what extent brain changes associated with addiction are more long-lasting than those related to other bad habits, or again falling in love. Here Lewis points to some recent research showing increases in grey matter after prolonged abstinence [20]. That is a nice and positive finding, but there are also numerous studies showing some return of function in some areas, but not in others, as recently reviewed [21]. In fact, many studies in this area report no full return of function after abstinence, because the abstinent ex-patients don’t reach normal levels of functioning. However, without a baseline measurement of functioning before the addiction, it cannot be ruled out that the suboptimal functioning may have been a premorbid factor, rather than an effect of the substance use [21]. Recent evidence not only points to excessive use as a cause of brain damage, but also to the number of detoxifications, which was shown to be related to functional damage in shifting in a reward task [22–24]. In summary, it seems fair to say that there can be some return of functioning after breaking with an addiction, but clearly there is also evidence for the opposite pattern, and this should be investigated further, ideally with baseline measures of functioning (which might happen with recent large scale longitudinal studies underway [25]).

A second argument is that the brain-disease perspective may be demoralizing for individuals. That may be true for some, and indeed there is some research pointing in this direction: for example, smokers who viewed smoking as an addiction had more troubles in quitting than smokers who viewed it as a bad habit [26]. When you believe you have lost your free will you can more easily shrug your shoulders, because you are actually not to blame. However, one could also argue the opposite (and in his introduction, Lewis points to this as well): the brain-disease perspective freed addicted people and their families from some of the social stigma related to the moral perspective on addiction. When addiction is a brain disease, immoral behaviours are less accountable than when it is the consequence of one’s immoral choices. But in the end this is not a central argument: the central question is whether addiction can best be seen as a brain disease or not, given the current state of the scientific knowledge. The secondary question is then how this is best transmitted and what the societal impact is of either message.

What is addiction then according to Lewis? Is it the same as any other result of our ever- learning and changing brain? Lewis proposes that addiction is a bad habit, which is in line with current neurobiological theories that emphasize the gradual change in addiction from motivated choice to compulsive behaviour, which is accompanied by a change in control from neural circuits involving the ventral striatum to circuits involving the dorsal striatum [6, 7]. The beautifully portrayed cases in Lewis’ book show that, contrary to popular belief, you can stop your addiction with enough will-power without any special treatment. And in fact, that’s what many people with addictions do. Just like when you fall in love, drug use is a motivated repetition that gives rise to deep learning. Desire is the number one emotional state that drives learning. Lewis then describes three specific mechanisms that make addiction exceptionally hard-to-overcome habits.

The first is a narrowed beam of attention for immediate reward and delay-discounting. According to Lewis this is exactly the state addicts find themselves in again and again. This is related to cognitive biases, which have indeed been related to addiction, and the good news is that they can be overcome through specific targeted training methods [27–30]. The second mechanism is that addictive rewards enlarge motivation. Our synaptic patterns get reinforced with every repetition (leaning a language, cooking, smoking) and the best way to do that is by boosting that repetition with strong motivation. Moreover, addictions have short-lasting rewards, and are often followed by negative affect, which can elicit the need for new stimulation. Or, in Lewis’ own poetic style: “Addictive rewards whet the appetite and leave frustration, loss and depression in their wake”. (ms. P. 11) Third, the addictive habit converges with other habits that thicken our personality, as in the crystallization of depressive or anxious traits. And indeed, there is evidence for increased impulsivity with adolescent addictive behaviour [5]. All in all, addiction is a habit that grows more quickly and becomes more deeply rooted in our neural circuitry than other habits, because of the intensity of the motivation of wanting to repeat them. This may sound a lot like a brain disease, but the difference is that Lewis’ perspective is the dynamic and developmental emphasis and the alternative remedy of further growth as the salivating answer, rather than repairing the “broken brain” in addiction as the disease model would suggest.

## “Trumping your Genes” and Motivation

What makes one individual more sensitive than another, when it comes to the development of addiction? There is little doubt in the scientific community that both genetic and environmental factors play an important role in individual differences in vulnerability for addiction [31]. What is interesting about Lewis’ position is that he takes people’s choices and conscious processing to be the ultimate cause of addiction and, vice versa, the ultimate cure of the plastic brain. His cases of Natalie, Brian, Donna, Johnny and Alice illustrate this. What this also tells us is that human agency is critically involved in the process. As the famous geneticist Kendler once put it in a lecture: you can have a lot of genetic risk factors increasing your vulnerability for addiction and still “trump your genes” by deliberately choosing to not start using an addictive substance when you know you are at risk (for example, because other family members developed an alcohol problem, you decide to never drink alcohol).

The motivation people gather to stop with their addiction makes use of the capacities of the mind. So, if addiction would be a brain disease where the capacity for *autonomous choice* is lost (in everyday language closely related to the philosophically difficult notion of “free will”), then the capacity to have the necessary clarity of mind to change the detrimental addictive behaviour could be gone. And that is exactly what Volkow and other proponents of the disease-model have proposed. [1, 9] So, the cases described in Lewis’ book make clear that even people suffering from severe addictions can sometimes have enough willpower to successfully change. In this positive scenario this mental capacity can function as an engine for positive change in the plastic brain, in line with the developmental model of Lewis. Similarly, Bill Miller argued that studying mechanisms in spontaneous recovery can help to develop effective interventions [32], and indeed, motivational interviewing, the therapeutic technique based on this insight, has been found effective in the treatment of addiction and beyond [33].

## Black Swans and a Gradual Model of Addiction

Although Lewis’ book is to be applauded for falsifying the idea that addiction is an incurable brain-disease, at the same time it is clear that not all cases of addiction are as rosy as the ones described in his book. In fact, addictions are among the most costly of all mental and brain disorders [34]. If, for example, Lewis had written a book on six cases of alcohol-dependence with Korsakoff’s syndrome (KS),^1^ a very different picture would have emerged: even if good resolutions are made, the affected brain is bound to forget them again [35].

It might be argued that the inclusion of KS as a consequence of alcohol addiction is problematic. One can have this condition through reasons not related to alcohol abuse, and not all people with alcoholism develop KS. However, we would argue that alcoholism is closely related to KS. In developed countries where people have a sufficient amount of thiamine in their diets, thiamine deficiency (the cause of KS) is almost always caused by alcoholism. [36] Regarding the reverse relationship: there are only a few estimates of how common KS is among alcoholics, but in autopsy studies, brain abnormalities characteristic of KS were present in about 13 % of alcohol-dependent patients. [37] Additionally, it is shown that most alcohol-dependent patients with cognitive impairment show at least some improvement in brain structure and functioning when abstinent for a time, meaning there is a definite link between alcoholism and cognitive ability. [38–40] Of course, this does not indicate that KS has a unique relation to alcoholism, but one could argue that the relationship with this brain-disease is far from trivial. Furthermore, other studies point to the fact that drug dependence can impair faculties of the brain that are crucial in recovering from addiction. [9, 22, 41] For instance, neuroimaging studies show an emerging pattern of generalized PFC dysfunction in drug-addicted individuals related to higher levels of drug use, worse PFC-related task performance and greater likelihood of relapse. [42]

We would argue that this shows that in some instances, severe drug and alcohol-dependence can damage the brain so severely that autonomous choice is largely lost. So from our perspective, the interesting empirical question would be who describes the white swans and who describes the black swans of addiction: is a brain disease the exception or the rule? Given that epidemiological data demonstrate that most cases of addiction cure without treatment, Lewis (and others like Heyman [43]), might be right that the rule is more that addictions are excessive cases of normal motivational mechanisms and habit formation [44], but, as argued above, there are also more severe cases, where the term brain-disease might be more appropriate. And the severe cases (often with lots of other problems) are more likely to seek professional treatment, because they did not manage to break the addiction without professional help. And indeed, the long-term outcomes of addiction-treatment are less positive, with a large majority relapsing within three years [45]. Hence, we think there is merit in the developmental model of addiction proposed by Lewis (and others [46, 47]), and that indeed, in many emerging cases in the population, the term “brain-disease” may be overly strong, but that in clinical populations there appear to be also cases where the term is justified. The interesting question is where to draw the line.

## What Do we Lose when we Are Addicted?

If addiction is a brain disease in which autonomous choice is threatened, it would be important to situate this claim within the debate on neuroscience and “free” will. To cite Wegner (2004, p. 657): “Most of us think we understand the basic issue of free will and determinism. The question seems to be whether all our actions are determined by mechanisms beyond our control, or whether at least some of them are determined by our free choice.” [48] However, it is important to distinguish between the actual mechanisms leading to action (which, as few will dispute, are produced by brain processes, given a learning history and genetic make-up), and the first-person feeling of free will. According to Wegner (2004), will is an authorship emotion, a somatic marker that authenticates the action’s owner as the self. This may or may not correspond to the actual causes, but it also serves a function: it allows us to maintain a sense of responsibility for our actions. In this sense, the disease view argues that autonomy (or its counterpart “free will”) may get lost in addiction, because the diseased brain determines the choices made in a reflexive way, given certain environmental (addiction-related) cues. The KS patient can make the resolution to quit drinking, but when next presented with a beer, will likely drink it. However, in many cases even people dependent on hard drugs have some ability left to choose other rewards over drugs, as Carl Hart had already elegantly demonstrated [49].

So what is this “autonomy” thing that appears to gradually and to some extent get lost in addiction? Most people hold a strong belief in free will: they think that what they want and will always causally determines their behaviour. This is one of the building blocks of civilization and law, but it is demonstrably wrong, as Wegner and others have demonstrated. [48, 50, 51] By now, many experiments in psychology and neuroscience have undermined this “naïve view” on free will/autonomy, and have shown that many aspects of our decision-making process are not as free as we think. First, the famous study of Libet demonstrated that neural signals that reliably predict a subjective feeling of choice appear about 350 milliseconds before the subjective experience of that same choice. [52–54] Second, Wegner and colleagues have demonstrated that you can carefully manipulate circumstances so that participants experience “free will”, even though this is ostensibly untrue. [50, 55, 56] Third, it has been shown that many other outside awareness may (partially) determine behaviour. [51, 57] This has led some neuroscientists (and “neurophilosophers”) to completely abandon the notion of free will, which from this perspective is only an illusion. The story is then that our conscious experience of agency and autonomy does not reflect actual agency, and in many cases it might mislead us. It shows that our non-conscious processes settle matters before we are ever aware that matters have been settled. It may seem like we guide our actions, but in reality we only experience our decision-making processes when it has already been decided. The research also shows that the common sense understanding of agency is misleading, because many elements that move us to act do so without our awareness of them. But that raises the question concerning what is lost or diminished in addiction, is that only an illusion [58]?

Clearly the debate on autonomy has not been settled yet. Libet’s results have been interpreted as evidence that neural processes determine our decisions well before we become aware, or these processes prepare a person’s decision before the person becomes aware of the preparations. Mele has argued against these strong interpretations of Libet’s findings [59], and argued that the results of the study are not strong enough to count the possibility out that a person can make a decision consciously. Libet’s findings depend on the assumption that the neural signals that reliably predict a subjective feeling of choice reflects preparation to decide. Schurger argued that we should rather see it as a reflection of neural noise that gradually increases in neural activity preceding spontaneous movements, but not in all cases of intentional movement [60]. The sceptical argument that our experience of acting is systematically illusory has been criticized; most notably Shepherd points out the fact that experiences of free will can be malleable doesn’t make them systematically illusory. [61] The studies that most threaten the belief that the human will is free are those purportedly showing that there are factors outside awareness that determine behaviour. Arguably, that only proves that the naïve perspective is incorrect, according to which “the feeling of free will” [50] always correctly indicates that our will determines our behaviour. Few people would dispute that, without buying into the notion that therefore autonomy is always illusory. In everyday life, many decisions are automatized, whether we drive a car, or engage in a conversation. Hence, it is interesting that sometimes the “feeling of autonomy” can be created, while people’s choices can be shown to be determined by external factors, but that does not prove that there is no role for willpower in human decision making.

In current psychological science and cognitive neuroscience, one position is in line with this criticism. In this view, conscious awareness and the feeling of autonomy are side effects of the unconscious processes that determine the choices made, but this conscious awareness does not have a direct causal effect in itself. However, they do have indirect effects: we have the ability to simulate non-existing hypothetical situations (or outcomes of possible choices), and these simulations may indirectly guide behaviour [62, 63]. This ability has been related to social communication and language [62], and to the ability to forego temptations [64].

One interesting theory was developed by Morsella, who argued that subjective states are necessary to coordinate different kinds of information in a complex modular brain, to foster adaptive behaviour. According to his supramodular interaction theory, adaptive behaviour in humans requires some supramodular response systems (defined by concerns). The instrumental system is concerned with interactions with the world. The incentive system is concerned with motivational orientation: should the organism approach or avoid a stimulus. Both systems can only interact in the subjective field. When you try to concentrate on the last pages of your book you really want to finish, it is possible to ignore your urge to go to the toilet, but sooner or later this becomes impossible or you will wet yourself: the competing incentive response system breaks into the subjective state. This interaction is necessary, because you can only perform one of the two behaviours at one moment. Therefore, subjective states are necessary to suppress the action tendencies of response systems. [65] Hence, there appear to be a set of neurocognitive functions which help us to steer our behaviour, overcome temptations, facilitate social interactions, that have sometimes been labelled “autonomy”. In severe cases of addiction, cue-elicited incentive processes may dominate the subjective state so strongly that indeed other signals cannot break through, as witnessed by wet pants, antisocial behaviours, and fatal accidents such as suffocating in vomit.

Even though the everyday naïve position on free will or autonomy (always right when we feel it) is incorrect, this is not to deny that these functions play an important role in our lives. One nice metaphor comes from Shariff and colleagues [63] concerning autonomy (and free will): we may subjectively feel as if we are operating a motorboat that we can directly steer to a goal, whereas in fact we may be operating a sailing boat that we steer more indirectly by adjusting the sails and keeping the long-term goal in consideration. And addictive behaviours can affect these functions, keeping the long-term goal in view, when there are distractions around.

### Back to Addiction

Both psychological faculties could be affected through addiction: (some of) the processes that actually determine behaviour which become biased to repeat the addictive behaviour) and/or the subjective feeling of free will. We believe that both could play a role; there is, for example, evidence that addiction makes one more myopic (weighing immediate consequences more strongly than later ones), which affects future behaviour (mechanism), but there is also evidence that the subjective idea that control is lost may affect future behaviour (apparent in cases of increased difficulty to quit smoking when one sees it as addiction as opposed to a bad habit). Most likely, the disease view will be primarily about the first possible type of consequence, related to the mechanisms underlying willed action: the ongoing desire for a certain substance takes a toll on autonomy and control. One interesting aspect of the debate is that here Lewis and Volkow appear to be on the same side: both recognize the importance of those mental functions which we describe under the vague notion of “free will” in overcoming addictive behaviours; the debate centers around what is left after addiction, and what is the norm. We argue that in most cases this will not be about black or white swans, but about grey geese.

This position is related to dual process models of addiction, which argue that in the non-addicted brain, there is a dynamic interplay between bottom-up impulsive processes (e.g., the slight smell of fire may be rightfully alarming, even though one is doing an exam), and top-down reflective processes [58]. One should note that dual system models have been criticized for being neurally implausible and theoretically fuzzy [66, 67]. As a solution, neurocognitive models have been proposed emphasizing temporal dynamics dependent on the reinforcement of cognitive functions together with generalizations of iterative re-processing, in which the features of cognitive-motivational processes shift from impulsive to reflective with more re-processing [68, 69], which also points to therapeutic interventions that weaken the influence of impulsive processes on behavior [27]. We would argue that this perspective naturally concurs with a more graded view on autonomy vs. brain disease in addiction.

## Conclusion

Free will, and the related concepts of autonomy, self-control or the ability to forego direct temptations for the benefit of more beneficial outcomes in the future, are dependent on mature brain functioning of circuits involving the frontal cortices and striatum. This ability develops in childhood [70], and individual differences in this ability have been related to long-term health outcomes, including addictions [71]. Moreover, these brain regions are affected by addictions, but does that imply that addiction is a brain disease? We argue for a more continuous perspective, where there are individual differences in these mental abilities (related to working memory capacity and other executive functions), and these abilities can be impaired through addictive behaviours, with a lot of individual differences in the extent to which this is the case, and the extent to which these abilities can recover [15, 21]. In extreme cases, with a lot of damage and little or no chance of recovery, the term brain disease may be in place, such as in alcohol-dependent patients suffering from KS. However, in most cases the term appears to be too extreme, and the more developmental dynamic perspective offered by Lewis and others may be more accurate. And as a positive by-product, this notion may be a more efficient engine for change in people affected by addictive behaviours.

## Footnotes

1 As it happens, a specialized clinic is close to his home, maybe the next book?
